# Fibroblast Growth Factor 23 in COVID-19: An Observational Study

**DOI:** 10.7759/cureus.42561

**Published:** 2023-07-27

**Authors:** Athena Myrou, Theodoros Aslanidis, Keli Makedou, Athanasios Mitsianis, Aikaterini Thisiadou, Paraskevi Karalazou, Georgios Chatzopoulos, Anastasios Papadopoulos, Antonios Kalis, Dimitrios Giagkoulis, Fotios Lezgidis, Christos Savopoulos

**Affiliations:** 1 Department of Internal Medicine, American Hellenic Educational Progressive Association (AHEPA) University Hospital, Thessaloniki, GRC; 2 Department of Intensive Care Unit, St. Paul Agios Pavlos General Hospital, Thessaloniki, GRC; 3 Department of Biochemistry, American Hellenic Educational Progressive Association (AHEPA) University Hospital, Thessaloniki, GRC; 4 Department of Internal Medicine, Mpodosakeio General Prefecture Hospital, Ptolemaida, GRC

**Keywords:** fgf23, clinical biomarker, infection, covid-19 infection, fibroblast growth factor 23

## Abstract

Introduction: Fibroblast growth factor 23 (FGF23) belongs structurally to the endocrine FGF protein family, which also includes FGF19 and FGF21. In the past decade, FGF23 has emerged as a possible diagnostic, prognostic biomarker, and therapeutic target in several conditions. Data about COVID-19 and FGF23 is still limited, yet they suggest interesting interactions.

Objective: In the present study, the levels of FGF23 were investigated in COVID-19 patients. These levels were also correlated with other inflammatory markers.

Materials and methods: In our prospective observational study, blood samples were collected from 81 patients admitted with COVID-19 (31 males and 50 females). We analyzed the relation of serum FGF23 levels with biochemistry, total blood count, coagulation parameters, and demographic data.

Results: The distribution of FGF23 serum levels according to sex and age (n_28-40_=8, n_41-60_=28, n_65-75=_ 25, n_75+_=20) was similar. No significant correlation between FGF23 and any other biochemistry, total blood count, and coagulation parameter was revealed in the whole sample. Nevertheless, there was a variation in the results among different age groups.

Conclusion: FGF23 levels seem to vary in symptomatic COVID-19 infection, but well-organized studies with larger numbers of patients in each group are needed to determine any reliable correlation between FGF23 and other laboratory parameters.

## Introduction

Fibroblast growth factor 23 (FGF23) belongs structurally to the endocrine FGF proteins family, which also includes FGF19 and FGF21. It is functionally considered a phosphatonin, a group of hormones that regulate phosphorus. Its prime target organ is the kidney; however, several tissues (bone tissue, bone marrow, vessels, ventrolateral thalamic nucleus, thymus, and lymph nodes) express FGF23 [1]. In the past decade, FGF23 has emerged as a possible marker (both diagnostic and prognostic) and therapeutic target in several conditions: hereditary diseases (syndromes of FGF23 excess and syndromes of FGF23 deficiency; hypophosphatemic and hyperphosphatemic disorders), acute renal failure and chronic kidney disease, stroke and subarachnoid hemorrhage, several types of neoplasms, psoriasis, gestational diabetes mellitus (DM), diabetic nephropathy, and bone metabolic disease and preclinical disease in type 2 DM [1-3].

Data about FGF23 levels and COVID-19 are scarce, yet interesting. A recent report of a small cohort of 149 patients due to SARS-CoV-2 infection found that blood serum proteins vary between asymptomatic and mild symptomatic infections in young adults, thus making them potential targets for developing new treatments (e.g., burosumab) and prognostic tests. It identified three immune mediators (interleukin 17C (IL-17C), matrix metalloproteinase 10 (MMP-10), and FGF23) that showed significantly higher levels in the asymptomatic group than in the early symptomatic group at the time of the first polymerase chain reaction (+) test and/or at 3-10 days after infection. Moreover, SARS- CoV-2 infection induced early IL-17C, MMP-10, FGF23, and chemokine ligand 23 upregulation in asymptomatic participants when compared to their baseline levels, while no significant changes (apart from a significant decrease of IL-17C) was detected in early symptomatic participants [4]. The immunoregulatory role of FGF23 has also been highlighted in COVID-19 and secondary immunodeficiency related to kidney disease [5].

Another review suggested that timely use of recombinant human erythropoietin (EPO), EPO analogs, acetylsalicylic acid, bioactive lipids, or FGF23 antagonists in genetically predisposed adults with the angiotensin-converting enzyme (ACE) D allele may induce detrimental effects in COVID-19 patients. In those individuals, this is attributed to ACE/ACE2 imbalance (triggered by ACE2 binding and internalization upon SARS-CoV-2 entry) that may lead to uncontrolled RAS overactivity and an angiotensin II (AngII)-induced pro-inflammatory state and immune dysregulation, These can provoke an increase in interleukin 6 (IL-6), plasminogen activator inhibitor, and FGF23. IL-6-induced EPO suppression, along with co-morbidities such as hypertension, diabetes, obesity, and RAS pharmacological interventions, may result in acute respiratory distress syndrome, cytokine storm, and/or autoimmunity [6]. In addition, a relationship between FGF23 and vitamin D administration in COVID-19 has been suggested [7].

The aim of the present study was to examine the levels of FGF23 in COVID-19 patients and to find possible relations with other, commonly used biomarkers.

## Materials and methods

In a prospective two-month observational study (AHEPA University Hospital IRB Approval No. 245/13.05.2021 ), blood samples of 90 adult patients admitted with COVID-19 were planned for further analysis after informed consent was obtained. Exclusion criteria included age of <18 years old, pregnancy, presence of other infection, severe condition (i.e., premortem or in shock, respiratory or any other organ failure that needs ICU monitoring and treatment) on admission, and the lack of informed consent.

Finally, data collected from 81 hospitalized patients admitted with COVID-19 (31 males and 50 females) aged 28-94 years were analyzed for biochemistry, blood count, and coagulation parameters (Table 1) along with FGF23 serum levels. Blood samples from nine patients were not included for further analysis due to protocol. Patients were distributed into the following age subgroups: n28-40=8, n41-60=28, n65-75=25, and n75+=20. Demographic data and comorbidities were also recorded. The two-tailed p-value of 0.05 was considered the significance threshold for all statistical tests. Data management and statistical analyses were conducted using MS Excel 2019 (Microsoft, Washington, USA).

**Table 1:** Laboratory parameters measured

IL-6	Interleukin-6	WBCs	White blood cells
CRP	C-reactive protein	Lymp%	% of lymphocytes
LDH	lactate dehydrogenase	Neu%	% of neutrophils
sUPAR	Soluble urokinase plasminogen activator receptor	Mono%	% of monocytes
fer	Ferritin	Eos%	% of eosinophils
PCT	Procalcitonin	Ba%	% of basophils
TnT	Troponin T	RBCs	Red blood cells
Glu	Serum glucose	Hgb	Hemoglobin
Ur	Urea	Hct	Hematocrit
Cr	Creatinine	MCV	Mean corpuscular volume
Urac	Uric acid	MCHC	Mean corpuscular hemoglobin concentration
SGOT	Aspartate transaminase	MCH	Mean corpuscular hemoglobin
SGPT	Alanine transaminase	RDW	Red cell distribution width
Alb	Albumin	RDW-SD	RDW-standard deviation
γ-GT	Gamma-glutamyltransferase	MPV	Mean platelet volume
CPK	Creatine kinase	PDW	Platelets distribution width
CPK-MB	CPK-MB isoform	p-LCR%	Platelet large cell ratio
Choles	Cholesterol	NRBC%	Nucleated RBCs
Tri	Triglycerides	MicroR	Micro-reticulocytes
HDL	High-density lipoprotein	MacroR	Macro-reticulocytes
LDL	Low-density lipoprotein	PT	Prothrombin time
ALP	Alkaline phosphatase	D-dim	D-dimers
Bil_tot_	Bilirubin total	aPTT	Activated partial thromboplastin time
Bil_dir_	Bilirubin direct	Fib	Fibrinogen
Bil_ind_	Bilirubin indirect	ESR	Erythrocytes sedimentation rate
Prot_tot_	Total serum protein	HbA1c	Glycated hemoglobin
Alb	Albumin	Ca	Serum calcium
Mg	Serum magnesium	Na	Serum sodium
P	Serum phosphorus	K	Serum potassium
PTH	Parathormone	TSH	Thyroid-stimulating hormone

## Results

**Figure 1:**
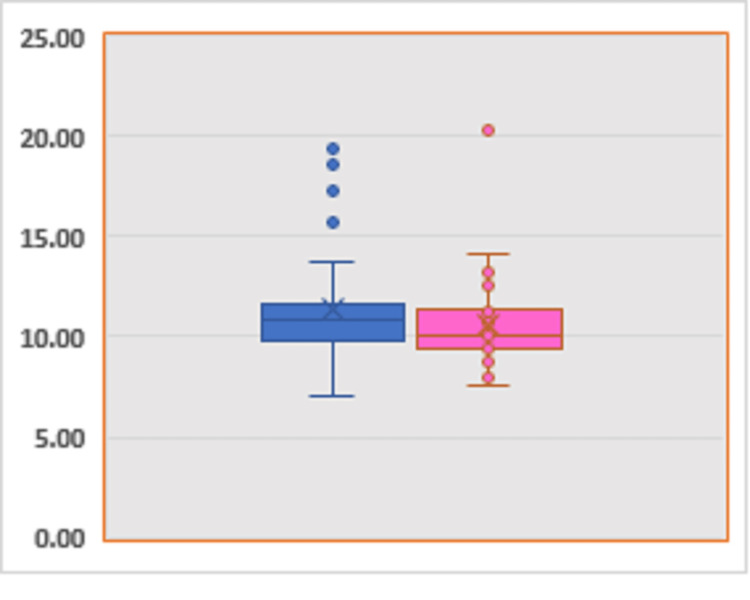
Distribution of FGF23 in male (blue) and female (pink) patients

**Figure 2:**
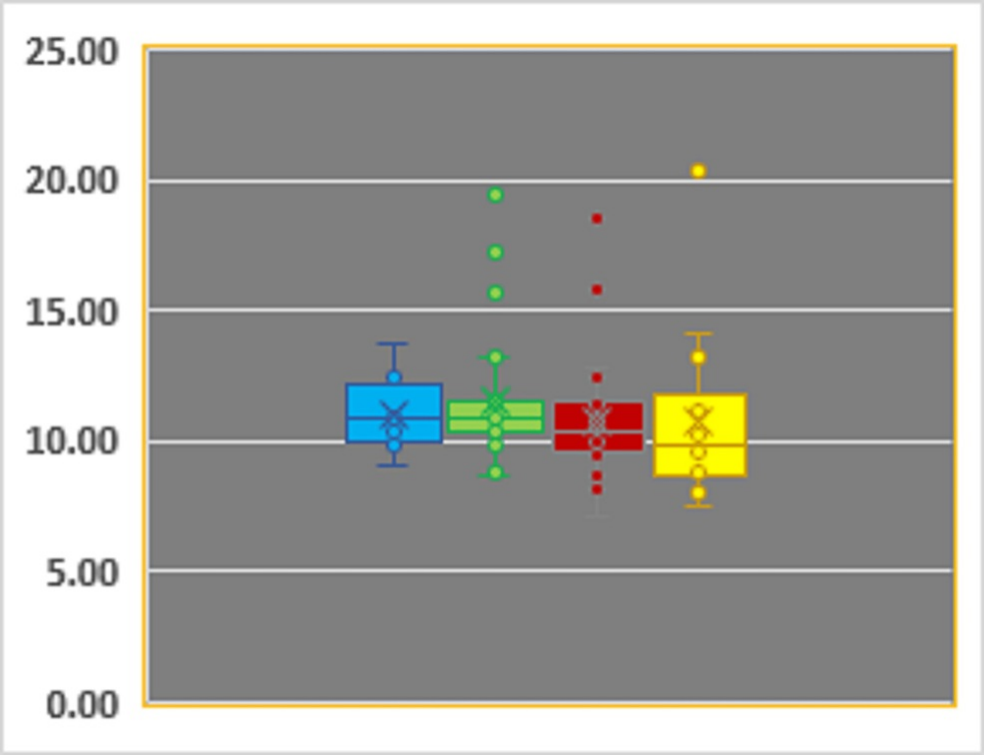
Distribution of FGF23 in patients younger than 40 (light blue), 40-60 (green), 61-75 (red), and 75+ (yellow) years old

The distribution of FGF23 values according to sex (31 males, 50 females) and age (n_28-40_=8, n_41-60_=28, n_65-75_=25, n_75+_=20) were similar (Figures 1-2).

Overall survival to discharge was 67.9%, whereas death incidence was almost equally distributed among sexes (men 55%/women 45%). Correlation exploration in the whole sample did not reveal any relation between FGF23 and any other parameter. However, further investigation did show moderate relations between the FGF23 and several other parameters in the group 28-40 years old. A weak relation was also observed in several other parameters, yet with small statistical significance (equal or p>0.05). The correlation coefficients (r^2^) are displayed in Τable 2.

**Table 2:** Correlation coefficients between FGF23 and several other parameters LDH: lactate dehydrogenase, WBC: white blood cell, fer: ferritin, PCT: procalcitonin, Ca: serum calcium, Alb: albumin, D-dim: D-dimers, PLT: platelet, TnT: troponin T, Glu: serum glucose, Ur: urea, SGOT: aspartate transaminase, γ-GT: gamma-glutamyltransferase, Choles: cholesterol, Tri: triglycerides, HDL: high-density lipoprotein, LDL: low-density lipoprotein, ALP: alkaline phosphatase, Bil_tot_: bilirubin total, Bil_dir_: bilirubin direct, Bil_ind_: bilirubin indirect, CPK: creatine kinase, CPK-MB: CPK-MB isoform, K: serum potassium, Na: serum sodium, P: serum phosphorus, Mg: serum magnesium, Prot_tot_: total serum protein, PTH: parathormone, TSH: thyroid-stimulating hormone, Neu%: % of neutrophils, Lymp%: % of lymphocytes, Mono%: % of monocytes, Eos%: % of eosinophils, Ba%: % of basophils, RBC: red blood cell, Hgb: hemoglobin, Hct: hematocrit, MCV: mean corpuscular volume, MCH: mean corpuscular hemoglobin, MCHC: mean corpuscular hemoglobin concentration, RDW: red cell distribution width, MPV: mean platelet volume, PDW: platelets distribution width, PCT PLT: procalcitonin platelet, p-LCR%: platelet large cell ratio, NRBC%: nucleated RBCs, MicroR: micro-reticulocytes, MacroR: macro-reticulocytes, PT: prothrombin time, INR: international normalised ratio, aPTT: activated partial thromboplastin time, Fib: fibrinogen, HbA1c: glycated hemoglobin, IL-6: interleukin-6, sUPAR: soluble urokinase plasminogen activator receptor, CRP: C-reactive protein, FGF23: fibroblast growth factor 23, FGF23m: men, FGF23f: women

	FGF23 age 28-40	FGF23 age 40-60	FGF23 age 60-75	FGF23 age75+	FGF23f	FGF23m
LDH	-0.492	0.012	-0.146	0.353	-0.034	-0.234
WBC	0.772	-0.092	-0.054	0.317	0.004	0.06
Fer	-0.564	0.118	0.364	0.191	0.48	-0.126
PCT	-0.439	0.188	-0.054	0.555	-0.037	0.045
Ca	0.817	-0.243	-0.37	-0.15	-0.336	0.379
Alb	0.232	-0.117	-0.152	-0.562	-0.197	0.21
D-dim	-0.239	0.036	-0.087	0.841	0.157	-0.272
PLT	0.649	0.106	-0.113	-0.245	-0.143	0.253
Tn T	0.626	-0.032	-0.051	-0.249	-0.014	-0.06
Glu	-0.0639	0.416	-0.205	0.166	-0.06	-0.278
Ur	0.197	-0.023	-0.021	-0.572	0.002	-0.101
Cr	0.274	0.108	-0.155	-0.435	-0.219	0.184
Urac	-0.207	-0.282	0.215	-0.56	-0.023	-0.316
SGOT	-0.435	-0.016	0.065	-0.026	0.045	0.041
γ-GT	-0.369	-0.086	0.014	-0.046	-0.033	0.074
Choles	0.311	0.402	-0.038	-0.458	-0.019	0.26
Tri	0.285	-0.082	-0.152	-0.305	-0.102	0.085
HDL	0.191	0.185	-0.359	-0.156	-0.257	0.128
LDL	0.779	0.233	0.073	-0.499	0.044	0.124
ALP	0.553	0.045	0.115	0.308	0.143	0.321
Bil_tot_	0.0764	-0.033	-0.015	0.323	-0.018	0.029
Bil_dir_	0.443	-0.038	-0.096	0.053	-0.047	0.112
Bil_ind_	-0.321	0.057	-0.201	0.69	-0.024	-0.105
CPK	0.684	-0.028	-0.251	0.207	-0.031	-0.169
CPK_MB	0.443	0.108	-0.139	-0.166	-0.102	0.43
K	0.324	0.045	-0.212	-0.075	-0.107	-0.257
Na	-0.318	0.112	0.032	-0.568	0.012	-0.307
P	-0.513	0.091	0.497	0.09	0.363	-0.085
Mg	0.743	0.195	0.123	-0.162	0.087	0.31
Prot_tot_	0.672	0.03	-0.183	0.077	-0.112	-0.12
PTH	-0.89	-0.161	0.15	-0.453	-0.078	0.051
TSH	0.255	-0.24	-0.069	-0.214	-0.1	-0.011
Neu%	-0.569	0.145	0.229	0.288	0.233	-0.003
Lymp%	0.015	-0.043	-0.103	-0.238	-0.126	-0.072
Mono%	0.39	-0.206	-0.093	-0.223	-0.118	0.192
Eos%	0.855	0.378	-0.087	0.031	-0.023	0.121
Ba%	0.132	0.181	-0.148	-0.338	-0.08	0.108
RBC	0.342	0.43	-0.202	-0.295	-0.112	-0.229
Hgb	-0.533	0.221	-0.006	-0.341	0.031	-0.173
Hct	-0.051	0.345	-0.009	-0.352	0.067	-0.183
MCV	-0.812	-0.077	0.077	0.044	-0.039	0.17
MCH	-0.813	-0.251	0.2	-0.129	0.114	0.079
MCHC	-0.834	-0.098	-0.077	-0.148	-0.189	0.016
RDW	0.154	-0.019	0.149	0.031	0.285	-0.043
RDW-SD		0.0286	0.337	-0.018	0.169	0.094
MPV		-0.208	-0.089	0.274	-0.051	-0.187
PDW	-0.279	-0.149	-0.09	0.347	-0.002	-0.278
PCT PLT		0.126	0.319	-0.189	-0.031	0.045
p-LCR%	-0.602	-0.147	0.234	0.335	0.206	-0.243
NRBC%	0.248	-0.187	0.443	-0.304	-0.068	-0.198
MicroR	0.74	-0.033	-0.093	0.0649	0.065	-0.235
MacroR	0.163	-0.006	0.102	-0.12	0.104	0.12
TKE	-0.281	-0.019	-0.066	-0.39	-0.079	0.156
PT	-0.296	-0.12	0.182	0.197	0.123	-0.027
INR	-0.048	-0.146	0.38	0.231	-0.007	-0.117
aPTT	-0.325	-0.319	0.024	0.053	-0.078	-0.046
Fib	0.485	-0.151	0.163	-0.116	0.077	0.204
HbA1c	-0.606	-0.123	-0.36	-0.054	-0.113	-0.577
IL6	0.215	0.182	-0.033	0.008	0.053	0.055
suPAR	0.104	0.168	-0.057	0.065	-0.017	0.132
CRP	-0.284	0.131	-0.072	-0.026	-0.035	-0.142

## Discussion

The present study suggests the differentiation of FGF23 levels with age, especially in younger ages and ages older than 75 years old.

In vitro and animal studies indicate that FGF23 acts as a deleterious agent by enhancing lung inflammation in various chronic lung diseases. Many of these effects are offset by klotho, a co-receptor for FGF23, which clearly protects the lung from inflammation. FGFR3/FGFR4 signaling contributes to the spatiotemporal specification of lung elastic fiber production [8]. However, it remains unclear how this tightly interacting FGF23/klotho pathway can be selectively targeted, which will be the key to developing new therapeutic interventions [9].

Other research has shown that high levels of FGF23 may have detrimental effects such as stimulation of inflammatory cytokines, left ventricle hypertrophy, and impairment of neutrophil function, thus affecting clinical outcomes and increasing mortality [10].

Earlier studies did not find any differences in total Galnt3 or Furin mRNA expression in their acute and chronic inflammation models while mentioning that chronic inflammation increases biologically active iFGF23 levels [11]. Their results suggest that increased activity of hypoxia-inducible factor (HIF) 1α is one mechanism through which inflammation stimulates FGF23 transcription. These data suggested that activation of HIF1α may be one mechanism that governs the coordinated response within osteocytes. FGF23 via prevention of β2-integrin activation inhibits the neutrophils' activation, binding, and migration and, thus, has been related to a higher prevalence of infections in ESRD patients [12].

Data about the presence of COVID-19 symptoms and levels of multiple inflammatory markers, though limited [4], strongly suggest their role, FGF23 among them, in the control of COVID-19 clinical signs. More specifically, IL-6 levels are significantly elevated and associated with adverse clinical outcomes in patients with COVID-19, playing a significant role in the phase of hyperinflammatory response [13]. Inhibition of IL-6 at the moment represents a therapeutic target for the management of dysregulated host responses in patients with COVID-19 and is recommended in international guidelines in cases of severe disease [14]. A recent experimental model has shown that IL-6 increases FGF23 expression and contributes to the high levels of FGF23 in uremic mice completing a positive feedback loop in which IL-6 increases FGF23 expression that in turn may promote inflammation [15]. This comes in accordance with previous work showing that FGF23 increases hepatic expression and secretion of inflammatory cytokines, contributing further to uremic inflammation [16]. It appears that FGF23 binds to hepatic FGFR4 and induces calcineurin/nuclear factor of activated T-cell signaling [16]. The latter leads to elevated expression of C-reactive protein and interleukin-6 [16-17].

In addition, FGF23 can induce the release of inflammatory cytokines from diverse reservoirs. In patients with chronic pulmonary disease, FGF23 levels are significantly increased [18-20]. Previous authors have shown that FGF23 acts upon cystic fibrosis human bronchial epithelial cells and leads to the secretion of IL-8, which is an important cytokine driving chronic inflammation in these patients [20-21]. Han et al. later revealed that FGF23 also targets peritoneal macrophages to increase the expression of TNF-α production, further expanding its role in inflammation [22].

Genomic0wide analysis data come to confirm the above studies, indicating that FGF23 regulates many proinflammatory genes [23]. FGF23-induced cytokine production is mainly due to NFAT activation, resulting in the induction of various cytokine genes like TNF-α, IL-2, IL-4, and IL-6 in distinct cell types such as T-cells and mast cells. It is, therefore, plausible that in view of the widely dysregulated inflammatory response that occurs in the setting of COVID-19, activation of "abnormal" FGFR4-dependent signaling in other cell types and tissues takes place [24-25].

Moreover, lung injury caused by COVID-19 has been found to activate IL-1 and NLRP3 inflammasome-mediated pathways contributing to the pathophysiology of COVID-19. IL-1α is primarily acting as an alarmin, following release by endothelial and epithelial cells, thus triggering NLRP3 inflammasome activation [26]. The latter leads to abundant IL-1β release and uncontrolled systemic hyperinflammation, resulting in pulmonary damage, as well as subsequent epitheliopathy and coagulopathy, reflecting widespread organ damage as this occurs in severe COVID-19 [27]. Recent data have come to support that systemic IL-1β seems to be the primary driver of FGF23 production [28], elucidating another pathway of interaction between inflammatory mediators and FGF23.

In cases of pulmonary fibrosis, FGFs play a role in tissue repair and the negative modulation of inflammation. More recent data suggest a relationship between FGF23 genetic polymorphism and renin-angiotensin-aldosterone system and FGF23 proinflammatory state, nucleotide-binding oligomerization domain (NOD)-like receptor protein 3 dysregulation, cytokine storm, the severity of COVID-19, and a multisystem inflammatory syndrome in children (MIS-C) with cardiac and/or enteric affliction [29].

The present study suggests the differentiation of FGF23 levels with age, especially in younger ages and ages older than 75 years old. However, these findings must be taken into account under several limitations. Though prospective, this is a single-center study with a small sample of recordings included. Larger studies with larger numbers of patients in each group are needed to determine any reliable correlation between FGF23 and other laboratory parameters. Another interesting perspective would be a time series analysis throughout the hospitalization of these patients. We also believe that the severity of the disease may provoke different and more profound changes.

Regarding relationships between 25(OH)D concentrations and COVID‐19 mortality, data are limited, and findings are mixed. That is the reason for not including vitamin D in the present study. Evidence documenting the role of vitamin D deficiency in the risk of infection by COVID-19 comes mainly from association studies, while in vitro or preclinical data regarding the protection provided by 1,25(OH)2D3 are extremely limited. Furthermore, despite the protective activity of 1,25(OH)2D3 in viral infections, there is evidence that its activity inhibits only certain viral infections, and the findings of its activity in in vitro infections of human cells by COVID-19 remain unreliable [30-31].

The majority of our experience exploring the association between plasma FGF23 levels, morbidity, and mortality from infectious causes comes from studies performed long before the SARS-CoV-2 pandemic and included infection-related hospitalizations caused by both bacterial and viral infections [32-33]. Recent evidence has come to show that FGF23 behaves as an immunomodulatory mediator, affecting the immune response, either by its direct actions on immune cells including neutrophils and macrophages, or indirectly on non-immune cells. Increased levels of FGF23 have been related to increased inflammation in patients [34] while inducing immune dysfunction in human white blood cells in vitro and in experimental models [12].

Evidence regarding the potential mechanisms pertaining to the association of high FGF23 levels and increased risk of severe infection remains elusive. FGF23 negatively regulates the 1-alpha hydroxylase, the enzyme responsible for active vitamin D synthesis (1,25 dihydroxy vitamin D), in renal, as well as extrarenal tissue, including monocytes [35-37]. A translational study showed that FGF23 per se is the cause of immune dysregulation in experimental models and immune cells recovered from healthy volunteers [12,38]. It appears that elevated FGF23 decreases neutrophil selectin-mediated rolling and chemokine-induced recruitment in vitro [38]. Moreover, the administration of FGF23 exacerbated disease and decreased survival, in experimental models of bacterial pneumonia in mice [12]. In this context, pharmacological blockage of FGF23 prevents cardiac and immune effects in vitro, although the exact underlying mechanisms have not been clarified yet. It is possible that FGF23 may cause indirect effects in other cells of the inflammatory milieu mediated by the activation of FGF receptors in neutrophil cells [39-40].

## Conclusions

FGF23 levels seem to vary in symptomatic COVID-19 infection, yet with distinct variation in different age groups. However, well-designed studies with a larger number of patients in each group are needed to determine any reliable correlation between FGF23 and other laboratory parameters. Other relationships with either severity or the stage of the infection and/or the comorbidities of each patient are yet to be clarified.
